# Neighbourhood deprivation is positively associated with detection of the ultra-high risk (UHR) state for psychosis in South East London

**DOI:** 10.1016/j.schres.2017.06.006

**Published:** 2018-02

**Authors:** V. Bhavsar, P. Fusar-Poli, P. McGuire

**Affiliations:** Department of Psychosis Studies, Institute of Psychiatry, Psychology and Neurosciences, King's College London, United Kingdom

## Abstract

**Background:**

Individuals are defined as being at ultra-high risk (UHR) for psychosis based on a combination of attenuated psychotic symptoms, help-seeking behaviour, genetic risk, and social/occupational deterioration. Limited evidence is available on whether UHR detection differs by neighbourhood, and potential explanations.

**Aims:**

To examine neighbourhood distribution of detected UHR using cases from the OASIS service in South East London, investigating neighbourhood deprivation as an explanatory variable.

**Methods:**

Geographic data were collected on patients who met UHR criteria over a fourteen-year period, at the neighbourhood (lower super output area, LSOA) level. Rates were calculated based on cases and age-specific population estimates. Poisson regression assessed associations between UHR rate and neighbourhood deprivation, and with particular deprivation domains, adjusting for referrals for UHR assessment, population density, and proportions of non-White people, and young single people.

**Results:**

Rate of UHR detection was statistically related to neighbourhood deprivation, but referral rate was not: compared to the least deprived neighbourhoods, the most deprived neighbourhoods had a greater than two-fold increase in incidence rate of detected UHR (adjusted incidence rate ratio (IRR): 2.11, 95% confidence interval (CI): 1.21,3.67). In contrast, a small, imprecise association was observed for referral for assessment for UHR (adjusted IRR: 1.26 (95%CI: 0.84,1.89)). Evidence was also found for associations of UHR detection rate with domains of deprivation pertaining to health and barriers to services.

**Conclusions:**

The distribution of UHR detection rates by neighbourhood is not random and may be explained in part by differences in the social environment between neighbourhoods.

## 1 Introduction

The ultra-high risk state (UHR) (Fusar-Poli et al., 2013b) for psychosis defines a group of people who are putatively at elevated risk for the development of psychotic disorders (Fusar-Poli et al., 2013a). At risk mental state services (Broome et al., 2005, Phillips et al., 2002) aim to intervene in this group with a view to preventing psychosis, thereby reducing the incidence and ensuing burden of these disorders at a population level. However, accessing these services requires help-seeking behaviour, which may not be commonplace in people with psychotic symptoms (Falkenberg et al., 2015, Fridgen et al., 2013, Green et al., 2011).

It is possible that the occurrence of the UHR state displays important variation by neighbourhood of residence (Oher et al., 2014), for three reasons. Firstly, it would be consistent with evidence for neighbourhood variation in the incidence of psychosis(Faris and Dunham, 1939, Kirkbride et al., 2016b, Pignon et al., 2016, Silver et al., 2002). For example, in a recent study based on data from early intervention services in the East of England, Kirkbride et al. (2016b) reported positive associations between psychosis incidence and both neighbourhood population density and neighbourhood deprivation. The most deprived neighbourhoods in the study had greater than double the rate of psychosis compared to the least deprived neighbourhoods (incidence rate ratio: 2.11; 95% confidence intervals (CI): 1.34, 3.32), after accounting for individual confounders that included ethnicity and occupational socioeconomic status. Secondly, neighbourhood variation in UHR would accord with emerging evidence that neighbourhood variation predicts general population sub-clinical psychotic experiences (Binbay et al., 2012, Das-Munshi et al., 2012, Schofield et al., 2016). Explanatory factors for this association include ethnic density (Boydell et al., 2001), social fragmentation (Kirkbride et al., 2008, Kirkbride et al., 2012), and neighbourhood deprivation (Bhavsar et al., 2014, O'Donoghue et al., 2016). Finally, neighbourhood variation in UHR might exist because help-seeking behaviour for mental health problems is itself associated with neighbourhood of residence, through mechanisms such as the geographic accessibility of services and proximity to alternative sources of support (Drukker et al., 2007b, Pickett and Pearl, 2001). However, whether neighbourhood variation exists in the UHR state, and whether this variation is related to underlying neighbourhood differences in help-seeking for mental health problems (Drukker et al., 2007a, Peterson et al., 2009), is not clearly established.

The UHR state is an important outcome for psychosis research – understanding the factors which influence the occurrence and detection of the UHR state, and which groups use early intervention services and which do not, is crucial for improving the effectiveness and efficiency of services. In this regard, evidence on neighbourhood variation could be important for targeting UHR-specific healthcare structures to specific types of area in order to improve their effectiveness, thereby assisting programmes aimed at the prevention of psychotic disorders at the population level (McGorry et al., 2008). Moreover, the occurrence of UHR might be more common neighbourhoods with higher levels of social deprivation, through mechanisms linked to stress processing and stress liability (Selten et al., 2013). Individuals who meet UHR criteria have an elevated risk for psychosis, and therefore understanding the factors that influence UHR risk could be important for aetiological research into psychosis itself.

In a previous study from our group which investigated associations between psychosis incidence and neighbourhood deprivation across different deprivation sub-domains (Bhavsar et al., 2014), we found that the association between psychosis incidence and neighbourhood deprivation was mainly accounted for by deprivation in the domains of crime and education, and sought to examine this further in relation to the UHR state. In this paper therefore, we investigate neighbourhood variation in the rate of positively detected UHR in South East London over a fourteen-year period (2002–2015). We compare this to the rate of referrals for UHR assessment in the same catchment area and timeframe, using regression models to evaluate neighbourhood deprivation and help-seeking as a possible explanation for variation in UHR detection rate. Finally, we explore which domains of deprivation may be most strongly predictive of UHR detection.

## 2 Methods

### 2.1 Description of sample

The initial dataset consisted of all help-seeking individuals assessed for suspicion of psychosis risk by the Outreach and Support in South London (OASIS) high-risk service, South London and Maudsley NHS Foundation Trust (SLaM). The OASIS service is a specialised service for the assessment and care of help-seeking people assessed to meet criteria for the UHR state. The service was established in 2001 by one of the co-authors (PM), in collaboration with others (Power et al., 2007). All individuals referred to OASIS during the period 1st January 2002–31st December 2015, and resident in either Lambeth or Southwark boroughs of the SLaM catchment area, were considered eligible. Subjects who were later found to already be psychotic by the time of proposed assessment were excluded. The remaining sample all undertook assessment for the UHR state using the CAARMS (Yung et al., 2006), a structured tool for the ascertainment of at-risk mental states. Assessments were carried out either by a psychiatrist or a psychologist, typically in pairs. Assessments took place at the OASIS team base clinic in South London. Each subject included in the study was therefore help-seeking, assessed using the CAARMS, and assigned either to a UHR positive or UHR negative category. Further details on the OASIS service (Fusar-Poli et al., 2013c), the assessment procedure (Yung et al., 2006), and the care provided within the service (Fusar-Poli et al., 2015), are published elsewhere.

### 2.2 Measurements

Information on age, gender, ethnicity and marital status was collected on all included subjects. Data on place of residence, in the form of postcodes (Gatrell, 1989), were used to group subjects into small geographic sectors, lower super output areas (LSOAs), the highest resolution of the spatial hierarchy for the reporting of neighbourhood statistics in the UK (ONS, 2015). Information was obtained from the UK Office for National Statistics (ONS) for all LSOAs located in Lambeth and Southwark on overall population, and population by single years of age, at the mid-points of the years comprising the study period (2002–2015) (ONS, 2017), and combined to estimate total person-years at risk for each LSOA over the course of the study. Data on deprivation for all LSOAs included in the study were obtained from the Office of the Deputy Prime Minister (ODPM, 2004). The Index of Multiple Deprivations 2010 reports continuous scores for seven domains of deprivation, which are employment deprivation, income deprivation, education deprivation, health deprivation, deprivation in living conditions, and barriers to housing and services, for each LSOA in England and Wales. These domains are based on census information relevant to each domain. Information on overall neighbourhood deprivation was taken from census data on the proportion of households exposed to more than one form of deprivation in each LSOA (ONS, 2011c). Data on proportion of non-white people (ONS, 2011a), proportion of single people between the ages of 16 and 36, and population density, in the form of persons per hectare (ONS, 2011b), for each LSOA were taken from census tables from the ONS, taking mid-points between census data on each variable for 2001 and 2011. Data on proportion of people who were non-White and proportion of young single people were re-scaled to improve interpretation of regression estimates - model coefficients for these variables therefore reflect change in the outcome for a 10% change in the value of each variable. Data on population density were also re-scaled for modelling, so that coefficients reflected change in rate for an increase in population density of one hundred persons per hectare.

### 2.3 Analysis

All statistical analyses were performed in STATA 14. Cases of UHR residing in LSOAs within Lambeth or Southwark were described for age, gender, ethnicity, marital status, and neighbourhood characteristics. Counts and rates for referrals and positively detected UHR in each LSOA were estimated. Associations were reported between rates of UHR in each LSOA for neighbourhood deprivation, and for each deprivation domain in turn, using Poisson regression. Population density measured by persons per hectare, proportion of non-white people, referral number and proportion of young (16–36) single people were included as covariates. Robust standard errors were used throughout. Likelihood ratio tests were used to assess evidence for a linear relationship between UHR detection rate and quintiles of deprivation.

## 3 Results

### 3.1 Sample size and description

Table 1 displays descriptive data on UHR-positive individuals. They numbered 336 in total, and were mainly male (*n* = 190, 57%). Included subjects had an average age of 23 (sd = 5.7). A greater percentage of UHR individuals lived in LSOAs in the most deprived quintile (22%) than the least deprived quintile (17%). The vast majority of cases (77%) were single and just under half (48%) were of White ethnicity (see Table 1). The estimate for the total person-years of observation for the study period was 2,347,022 person-years, giving an overall UHR detection rate of 14.3 cases per hundred thousand person-years (95%CI: 12.8,15.9), and an overall referral rate of 26 referrals per hundred thousand person-years (95% CI: 24.0, 28.2).

**Table 1:** Description of individuals defined as meeting ultra-high risk criteria.

			Mean (sd)	Count (%)
				–
Neighbourhood (LSOA) characteristics	Proportion of multiply deprived households		0.20(0.05)	
	Employment deprivation		0.13(0.94)	–
	Income deprivation		0.11(0.94)	–
	Education deprivation		0.04(0.96)	–
	Health deprivation		0.19(0.87)	–
	Living standards deprivation		0.15(0.99)	–
	Barriers to housing/services		− 0.07(0.81)	–
	Crime deprivation		0.02(0.99)	–
	Proportion(%) of non-White people		42.2(12.77)	–
	Proportion(%) of young single people		81.1(5.07)	–
	Population density (persons per hectare)		133.66(52.62)	–
	Neighbourhood deprivation^a^			
	1 (least deprived quintile)		–	56(17)
	2 (second least quintile)		–	73(22)
	3 (intermediate quintile)		–	62(18)
	4 (second most deprived quintile)		–	71(21)
	5 (most deprived quintile)		–	74(22)
Individual characteristics	Age		23.13(5.71)	–

	Gender	Female	–	146(43)
		Male	–	190(57)
	Marital status	Married/in a relationship	–	20(6)
		Divorced/separated	–	12(4)
		Single	–	259(77)
		Missing	–	45(13)
	Ethnicity	Black	–	105(31.3)
		White	–	160(48)
		Any other	–	44(13)
		Asian	–	12(4)
		Missing	–	15(4)
		Grand Total	–	336(100)

a Refers to LSOA in which individual lived at time of assessment by OASIS.

### 3.2 Neighbourhood deprivation and the rate of UHR and of referrals

The incidence rate of positively detected UHR, and of referral for UHR assessment, was progressively higher across increasingly deprived LSOAs, based on quintiles of proportion of multiply deprived households. The median LSOA rate of UHR in each quintile also increased with increasing quintile of deprivation (see Table 2), however this pattern was not observed for referrals for UHR assessment. The rate of referral for UHR assessment in each quintile of deprivation ranged from 25.6 referrals per hundred thousand person-years in the least deprived quintile to 30.3 referrals per hundred thousand person-years in the most deprived quintile. The rate of positively detected UHR in each quintile of deprivation ranged from 9.1 cases per hundred thousand person-years in the least deprived quintile to 19.8 cases per hundred thousand person-years in the most deprived quintile.

**Table 2:** Descriptions of LSOAs by quintiles of neighbourhood deprivation based on 328 LSOAs.

Neighbourhood deprivation	Referrals for UHR assessments	UHR cases	Person-years at risk	Rate of UHR in quintile per hundred thousand person-years	Rate of referral in referrals per hundred thousand person-years	Median rate of UHR in cases per hundred thousand person-years (number of LSOAs)	Median rate of referral in referrals per thousand person-years (number of LSOAs)
1 (least deprived quintile)	124	44	483,532	9.1(6.6,12.2)	25.6(21.3,30.6)	9.7(62)	20.3(62)
2 (second least deprived quintile)	118	67	508,669	13.2(10.2,16.7)	23.2(19.2,327.8)	12.0(69)	17.4(69)
3 (intermediate quintile)	107	76	478,887	15.9(12.5,19.9)	22.3(18.3,27.0)	11.7(66)	17.9(66)
4 (second most deprived quintile)	128	73	434,549	16.8(13,21.1)	29.5(24.6,35.0)	14.4(64)	21.6(64)
5 (most deprived quintile)	134	76	441,385	19.8(13.6,21.6)	30.3(25.4,36.0)	15.2(67)	24.6(67)

In univariate analyses (Table 3), a positive association was observed between UHR detection rate and neighbourhood deprivation – LSOAs in the highest quintile of deprivation had a URH detection rate 1.97 times higher than LSOAs in the least deprived quintile (IRR 1.97, 95%CI: 1.38,2.82). Association was also observed between UHR detection rate and increasing proportion of non-white people (IRR for a ten percentage point increase in non-white people: 1.10 (95%CI: 1.02,1.19). When all neighbourhood characteristics were entered into a fully-adjusted model, only neighbourhood deprivation remained associated with UHR detection rate, after adjusting for non-white people, proportion of young single people, population density, and referrals for UHR assessment (p for linear trend = 0.012). The most deprived LSOAs experienced more than twice the rate of UHR detection compared to the least deprived LSOAs (IRR: 2.11; 95%CI: 1.24,3.59). In contrast, there was a small, statistically imprecise association between neighbourhood deprivation and rate of referral for UHR assessment- after adjustments, the most deprived neighbourhoods experienced 1.26 times the rate of referral for UHR assessment compared to the least deprived neighbourhoods (*p* = 0.217) (Table 4).

**Table 3:** Estimates for the association (IRR) between positive UHR detection and neighbourhood (LSOA) variables. All estimates based on 328 LSOAs.

	Unadjusted IRR(95%CI)	P	Fully-adjusted IRR (95%CI)	P
Neighbourhood deprivation				
Least deprived quintile	Reference		Reference	
Second least deprived quintile	1.44(0.98,2.10)		1.44(0.97,2.15)	
Intermediate quintile	1.69(1.12,2.56)		1.69(1.09,2.62)	
Second most deprived quintile	1.89(1.27,2.80)		1.91(1.12,3.26)	
Most deprived quintile	1.97(1.38,2.82)	<* 0.001* (trend)	2.11(1.24,3.59)	*0.012* (trend)
Ten per cent change in proportion of non-White people	1.10(1.02,1.19)	*0.011*	1.02(0.89, 1.17)	0.808
Ten per cent change in proportion of young single people	4.59 (0.52,40.74)	0.171	9.55(0.96,95.30)	0.055
Population density (hundred persons per hectare)	1.07(0.84,1.36)	0.583	0.90(0.68,1.20)	0.480
Referrals	1.05(0.99,1.10)	0.099	1.03(0.98,1.09)	0.260

*P*-values are reported from Wald tests, and are italicised where they fall below the pre-defined alpha-level (0.05). Fully-adjusted models included non-White proportion, young single proportion, population density, and referral number.

**Table 4:** Estimates for the association (IRR) between referral for UHR assessment and neighbourhood (LSOA) variables. All estimates based on 328 LSOAs.

	Unadjusted IRR (95%CI)	P	Fully-adjusted IRR (95% CI)	P
Neighbourhood deprivation				
Least deprived quintile	Reference		Reference	
Second least deprived quintile	0.84(0.64,1.11)		0.85(0.64,1.13)	
Intermediate quintile	0.82(0.64,1.04)		0.84(0.64,1.09)	
Second most deprived quintile	1.12(0.86,1.45)		1.15(0.85,1.56)	
Most deprived quintile	1.19(0.84,1.67)	0.102 (trend)	1.26(0.84,1.89)	0.217 (trend)
Ten per cent change in proportion of non-white people	1.04(0.98,1.12)	0.235	1.02(0.97, 1.08)	0.458
Ten per cent change in proportion of young single people	0.88(0.19,4.02)	0.870	1.34(0.26,6.84)	0.722
Population density (hundred persons per hectare)	0.91 (0.77,1.08)	0.262	0.82(0.70,0.97)	*0.018*

*P*-values are reported from Wald tests, and are italicised where they fall below the pre-defined alpha-level (0.05). Fully-adjusted models included non-White proportion, young single proportion, and population density.

Crude associations were observed between the UHR detection rate and deprivation domains related to employment, income, education, and health. On adjustment for all covariates, good statistical evidence remained for positive association between UHR detection rate and health deprivation, and a negative association with barriers to housing and services (see Table 5).

**Table 5:** Univariate associations between rate of UHR detection in LSOAs and each domain of deprivation. All estimates based on 328 LSOAs.

Neighbourhood deprivation domain	Model I: unadjusted IRR (95% CI)	Model II: IRR (95% CI) from model II adjusted for population density	Model III: IRR (95% CI) from model II further adjusted for referrals	Model IV: IRR (95% CI) from model III further adjusted for proportion of single people, and proportion of non-White people
Employment deprivation^a^	1.19(1.07, 1.33)	1.20(1.08, 1.33)	1.19(1.07,1.33)	1.15(0.98, 1.37)
Income deprivation^a^	1.16(1.05, 1.29)	1.18(1.06, 1.31)	1.17(1.05, 1.30)	1.11(0.99, 1.10)
Education deprivation^a^	1.14(1.01, 1.28)	1.14(1.00, 1.28)	1.14(1.01, 1.29)	1.09(0.95, 1.26)
Health deprivation^a^	1.20(1.07, 1.35)	1.20(1.07, 1.35)	1.20(1.07, 1.35)	1.20(1.07, 1.35)
Living standards deprivation^a^	1.04(0.92, 1.19)	1.04(0.91, 1.18)	1.03(0.90, 1.17)	1.02(0.89, 1.16)
Barriers to housing/services^a^	0.93(0.84, 1.04)	0.92(0.82, 1.04)	0.92(0.81, 1.03)	0.81(0.69, 0.97)
Crime deprivation^a^	0.99(0.87, 1.13)	1.00(0.87, 1.14)	0.99(0.87, 1.13)	0.97(0.86, 1.10)

Incidence rate ratios estimated by Poisson regression with robust variances.

a All scores for neighbourhood conditions are z-standardized.

## 4 Discussion

### 4.1 Summary of findings

The positive association between UHR detection rate and neighbourhood deprivation remained after adjustment for population density, proportion of young single people, proportion of non-White people, and for referrals for UHR assessment. In contrast, there was a small and imprecise association between neighbourhood deprivation and rate of referral for UHR assessment. Crude associations were observed between UHR detection rate and deprivation in income, employment, education, and health, however on adjustment for other possible influences on UHR detection rate, only the association with health deprivation retained good statistical evidence. A negative association, not present in unadjusted models, was observed between UHR detection rate and barriers to housing and services.

### 4.2 Previous literature

Important neighbourhood variation in the occurrence of psychotic disorders has been suggested for some time (Faris and Dunham, 1939, Silver et al., 2002). There is now evidence for neighbourhood variation in the occurrence of sub-clinical psychotic symptoms(Schofield et al., 2016) and symptoms of psychosis in people with established psychotic disorders(Oher et al., 2014). A small number of studies of UHR and neighbourhood deprivation have suggested a positive association, however studies have been limited by small sample sizes, and limited adjustment for neighbourhood confounders. O'Donoghue et al. (2015) reported that the rate of UHR identification in neighbourhoods with above average levels of deprivation was 1.51 times higher compared to neighbourhoods with the least deprivation (rate ratio 1.51,95%CI: 0.93,2.53), but were not able to account for differences between individuals. This association was not statistically significant, and no association was found for the highest level of deprivation. Kirkbride et al. (2015) carried out a multi-level analysis accounting for individual characteristics and found differences in the spatial distribution of UHR compared to controls, and reported that this distribution was predicted by deprivation, ethnic density, and proportion of single-parent households; however, after all adjustments, a negative association with deprivation was found, with individuals from neighbourhoods with greater proportion of deprived households experiencing a relative reduction in the odds of UHR (OR:0.86,95%CI:0.75,0.98). These mixed findings might be explained by an underlying non-linearity in the relationship between neighbourhood deprivation and detection rate for UHR (Kirkbride et al., 2016a); however in our analysis we found good evidence for a linear association between UHR detection rate and neighbourhood detection.

In contrast to previous work, we used multivariable regression models to assess whether the association with UHR was explained by the influence of other neighbourhood characteristics, including proportion of young single people, population density, and proportion of non-White people. To our knowledge, previous studies have not investigated the role of spatial patterning of help-seeking behaviour as an explanation for the association between neighbourhood deprivation and UHR detection rate- our results suggest that neighbourhood patterning in the detection rate of UHR is not explained by differences in help-seeking behaviour between neighbourhoods. We also tentatively identified potential explanations for the association with deprivation by looking at different domains of deprivation. The negative association with barriers to housing and services (a marker of proximity to primary care services, schools and supermarkets), despite adjustment for referrals, implies that the positive detection of UHR could be related to the geographic accessibility of services in neighbourhoods, and requires further investigation. The association with health deprivation, a dimension reflecting morbidity, mortality, disability, and the occurrence of mood and anxiety disorders, is consistent with common ecological correlates with a broad range of health problems (Pickett and Pearl, 2001).

### 4.3 Strengths and limitations

Although this study examined UHR detection over fourteen-year period, from a UHR service covering a large catchment area, with access to information on deprivation and area of residence, the analysis nevertheless has limitations. This was an ecological study, assessing the association between the neighbourhood rate of UHR and neighbourhood deprivation. Therefore, our results are not able to directly interrogate whether neighbourhood deprivation is a causal factor for the development of UHR at the individual level (Susser, 1994), as would be possible in a multi-level study (Kirkbride et al., 2015). However, the association reported here does permit conclusions concerning the allocation of early intervention resources to more deprived areas, and the relative importance of help-seeking behaviour in explaining this association. Population estimates for LSOAs were taken from the UK ONS, and were based on statistical models that may have been mis-specified (Bates, 2005). Although other neighbourhood factors could have more explanatory power for the rate of UHR, the neighbourhood variables used in this study are widely used in health research on other outcomes (Fox et al., 2011, Lang et al., 2008a, Lang et al., 2008b), and are based on a large range of census data. Poisson regressions for the main effects were specified based on the prior hypotheses, but overall models fits were poor, although associations had reasonable statistical evidence. Furthermore, this analysis did not take account of measurement misclassification in the outcome – individuals may have been incorrectly classified as UHR when they were in fact not. This mechanism might have introduced bias, if this misclassification were to have been related to deprivation level- that is, were individuals from more deprived neighbourhoods been more likely to be classified as UHR when they were, in fact, not, then this would have overestimated the rate of UHR in more deprived areas. However, there is little evidence to suggest systematic misclassification of UHR based on where the patient lives.

### 4.4 Concluding remarks

We found that UHR detection appears to be more common in more deprived neighbourhoods, consistent with the wider psychosis literature. This was not fully explained by spatial inequality in referral to UHR services, or by differences in ethnic composition of areas, or by population density. Neighbourhood associations are likely to be explained primarily by differences in the composition of areas, and any truly contextual effects, for example related to the experience of deprived areas as threatening/defeating (Selten and Cantor-Graae, 2007), are likely to be small (Diez Roux, 2001). However, our findings suggest that resources for UHR detection could be meaningfully directed into more deprived communities, resulting in potential improvements in the efficiency and effectiveness of psychosis early intervention detection and intervention strategies for the UHR state for psychosis.

## Contributors

VB designed, conducted, and wrote the first draft of the analysis. PFP was responsible for overseeing the collection of data and preparing the data for analysis. PM and PFP contributed to interpreting the results and writing the final paper.

## Role of the funding source

Vishal Bhavsar is supported by a Wellcome Trust Clinical Research Training Fellowship in Epidemiology (101681/Z/13/Z). The funder had no other role in the research.

## Conflicts of interest

The authors have no conflicts of interest to declare.
